# A large dataset of semantic ratings and its computational extension

**DOI:** 10.1038/s41597-023-01995-6

**Published:** 2023-02-23

**Authors:** Shaonan Wang, Yunhao Zhang, Weiting Shi, Guangyao Zhang, Jiajun Zhang, Nan Lin, Chengqing Zong

**Affiliations:** 1 National Laboratory of Pattern Recognition, Institute of Automation, CAS, Beijing, China; 2 School of Artificial Intelligence, University of Chinese Academy of Sciences, Beijing, China; 3 CAS Key Laboratory of Behavioural Sciences, Institute of Psychology, Beijing, China; 4 Department of Psychology, University of Chinese Academy of Sciences, Beijing, China

**Keywords:** Human behaviour, Language

## Abstract

Evidence from psychology and cognitive neuroscience indicates that the human brain’s semantic system contains several specific subsystems, each representing a particular dimension of semantic information. Word ratings on these different semantic dimensions can help investigate the behavioral and neural impacts of semantic dimensions on language processes and build computational representations of language meaning according to the semantic space of the human cognitive system. Existing semantic rating databases provide ratings for hundreds to thousands of words, which can hardly support a comprehensive semantic analysis of natural texts or speech. This article reports a large database, the Six Semantic Dimension Database (SSDD), which contains subjective ratings for 17,940 commonly used Chinese words on six major semantic dimensions: vision, motor, socialness, emotion, time, and space. Furthermore, using computational models to learn the mapping relations between subjective ratings and word embeddings, we include the estimated semantic ratings for 1,427,992 Chinese and 1,515,633 English words in the SSDD. The SSDD will aid studies on natural language processing, text analysis, and semantic representation in the brain.

## Background & Summary

Accumulating behavioral and neural evidence indicates that semantic representation of words is distributed across multiple neural subsystems, each representing a particular dimension of semantic information^1,2^. These semantic subsystems and dimensions provide important clues for the organization of the human semantic system. To investigate how word meaning is represented and processed in the human brain, many studies have built databases of word ratings on the psychologically and neurobiologically plausible semantic dimensions^1,3–6^. Different from free-association-based^7^ and feature-generation-based^8,9^ semantic databases, dimension-based semantic databases provide quantified rating scores for words on experiential semantic dimensions, which enable investigating the behavioral and neural impacts of semantic dimensions on language processes^3,4,10^ and building computational representations of language meanings^11–15^. However, the existing databases typically contain hundreds to thousands of words and are not large enough to support comprehensive semantic analysis of natural texts or speech. For example, if a researcher wants to analyze the behavioral or neural effects of particular semantic dimensions of words during natural text processing, then they may want to conduct an analysis using the semantic ratings of all or most of the words of the text; however, in most cases, the existing semantic rating databases can only provide the ratings of a small part of the words.

This article reports a large semantic rating database named the Six Semantic Dimension Database (SSDD)^16^. The SSDD focuses on six major semantic dimensions: vision, motor, socialness, emotion, time, and space. The visual and motor dimensions are included to reflect the impact of sensory-motor experience on semantic representation. Sensory and motor dimensions are probably the most frequently investigated semantic dimensions, and their importance for object and action concepts has been well established^1,4,17–20^. Among the multiple sensory dimensions associated with semantic representation, we chose the visual dimension because vision is the dominant sensory modality. The behavioral and neural impacts of visual and motor semantics on cognitive processing have been indicated by many previous studies^18,19,21–25^. The social and emotional dimensions are included to reflect the impact of social-emotional experience on semantic representation. These dimensions have dissociable neural correlates^10,24,26–33^ and are especially important for the representation of mental and abstract concepts^5,32–35^. Huth *et al*.^2^ investigated the organization of semantic representation in the brain using a data-driven approach and found that social-emotional and sensory-motor semantics are associated with the opposite ends of the most important data-driven semantic dimension. Therefore, the social and emotional dimensions can serve as important supplements to the visual and motor dimensions to reflect semantic representation. The time and space dimensions are especially important for the representation of events and situations^36–38^. Dissociable neural correlates of these dimensions have also been indicated by neuropsychological and neuroimaging research^37,39^. The representativeness of the six dimensions has been reflected by a comprehensive review of experiential semantic attributes by Binder *et al*.^1^. Binder *et al*.^1^ summarized 65 semantic dimensions belonging to 14 domains, among which more than 2/3 of the dimensions belong to the domains of vision, motor, socialness, emotion, time, and space. The SSDD treats these six domains as coarse-grained semantic dimensions and provides general ratings for each of them.

The SSDD contains two datasets: the first is the subjective ratings for 17,940 commonly used Chinese words on the six semantic dimensions. The second is a computational extension of the subjective rating data. We combined the subjective ratings with computational models and then estimated the semantic ratings of 1,427,992 Chinese and 1,515,633 English words. The SSDD makes it possible to analyze the semantic components of various natural language materials, such as natural texts, speeches, and the language produced by neurological and psychiatric patients.

## Methods

### Subjective rating dataset

#### Participants

A total of 85 healthy undergraduate and graduate students (52 women, M age = 22.73 years, SD age = 2.24) participated in the rating experiment. All participants were native Chinese speakers. No participant had suffered from psychiatric or neurological disorders or sustained a head injury. Each participant read and signed the informed consent form before the experiment. All experiments were approved by and performed in accordance with guidelines and regulations of the Institutional Ethics Committee at the Institute of Psychology of the Chinese Academy of Sciences. Participants were asked to complete at least one rating experiment session (see Procedure of the rating experiments) and were compensated with 30 RMB per session. Each participant could complete as many sessions as they wanted as they passed the quality evaluation every time. Those who failed the quality evaluation once were not allowed to complete more sessions. Following exclusions (see Procedure of the rating experiments), the final sample comprised 80 participants (49 women, M age = 22.88 years, SD age = 2.21) who provided at least one session of valid data.

#### Stimuli

The stimuli were 17,940 items that could be separated into three sets based on their sources. The first set of items was 12,814 high-frequency Chinese words selected from the Wikipedia Chinese corpus (https://dumps.wikimedia.org/zhwiki/latest/zhwiki-latest-pages-articles.xml.bz2). These items were selected based on four inclusion criteria: (1) They are the 20,000 most frequent items of the Wikipedia Chinese corpus; (2) they are also included in at least one of two supplementary Chinese corpora, that is, the Contemporary Chinese Dictionary^40^ and the Chinese Linguistic Data Consortium (2003) corpus (https://catalog.ldc.upenn.edu/LDC2003T09); (3) they do not contain any non-Chinese characters; and 4) they were judged as Chinese words but not phrases or nonwords and were not judged as proper nouns by at least two of three independent raters (two authors, Nan Lin and Weiting Shi, and a graduate volunteer). We used supplementary Chinese corpora and subjective assessments because the boundaries between words and phrases in Chinese are vague. There is often a discrepancy between different corpora and between corpora and subjective judgments^41^. We excluded proper nouns because participants’ knowledge of them may highly depend on personal experiences and interests.

The second set of items was 4,915 Chinese words selected from the stimuli of two recently published fMRI datasets^42,43^, a published study^10^, and several unpublished experiments of ours. Items were excluded if they contained non-Chinese characters or were evaluated as nonwords, phrases, or proper nouns by at least two of three independent raters.

The last set of items was 211 Chinese translations for the English stimuli of the semantic rating experiments from Binder *et al*.^1^ and Tamir *et al*.^5^. The two studies included 535 and 166 English words, respectively. Most of their Chinese translations had already been included in the first two sets of items. The remaining 211 translations (which include a small number of phrases) were included as the last set of items. The rating data of these items were used to validate the results (see Technical Validation).

#### Procedure of the rating experiments

We conducted six rating experiments on the 17,940 items, each focusing on one semantic dimension. Each experiment was separated into 18 sessions containing 1,000 words (the last session contained 940 words). The data were collected through the free-access online platform “Wen Juan Xing” (https://www.wjx.cn/). Except for the rating experiment on the semantic dimension of emotion, which used a 13-point scale (−6 = very negative, 0 = neutral, and 6 = very positive), all other rating experiments used 7-point scales (7 = very high, and 1 = very low). Before each rating session, participants read instructions about the working definitions for the semantic dimension to be rated (See Table 1) plus a few example words with high and low ratings. For the semantic dimension of motor, we further specify the working definition based on the charade/pantomime rating from previous studies:^18,44–46^ “Please rate the extent to which the meaning of a word can easily and quickly trigger corresponding body actions in your mind. Specifically, suppose you were playing a pantomime game in which one person had to identify a word based on how another person mimicked various actions that might be associated with its meaning. The easier a word is for the game, the higher its rating score should be; the harder a word is, the lower its rating score should be.”

**Table 1:** Working definition of each semantic dimension.

Dimension	Working definition
Vision	the extent to which the meaning of a word can easily and quickly trigger corresponding visual images in your mind
Motor	the extent to which the meaning of a word can easily and quickly trigger corresponding body actions in your mind
Socialness	the extent to which the meaning of a word relates to relationships or interactions between people
Emotion	the extent to which the meaning of a word relates to positive or negative emotions
Time	the extent to which the meaning of a word relates to time, including early or late, length, sequence, frequency, etc.
Space	the extent to which the meaning of a word relates to spatial information, including location, direction, distance, path, scene, etc.

To control the quality of the rating data, after each session of rating, we calculated the correlation between the ratings of each participant and the mean ratings of the remaining participants using Jamovi (https://www.jamovi.org/). For a given session, if the correlation between the ratings of a participant and those of the others was lower than 0.5, then the data of this participant would be excluded^1^, and the participant would be excluded from the rest of our experiment. This criterion resulted in the rejection of 28 sessions or 0.87% of the data. If the data of a participant were excluded, a new participant was recruited to complete the rating session. For each session of each experiment, 30 valid participants were recruited.

#### Data analysis

For each experiment, we calculated the average rating for each word to represent its value on the rated semantic dimension. In addition to the six rated dimensions, a seventh semantic dimension was obtained by calculating the absolute value of the average emotion rating for each word. We believe this dimension (i.e. valenced vs. neutral) reflects the relatedness of word meanings to emotion (see Technical Validation for evidence of this argument). We added 1 to this measure to match its scale with that of the five nonemotion ratings.

**Fig. 1:**
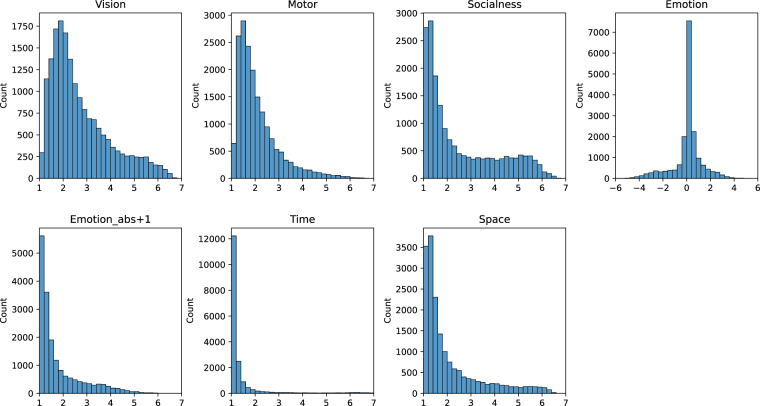
Distribution of ratings for the seven semantic dimensions.

**Fig. 2:**
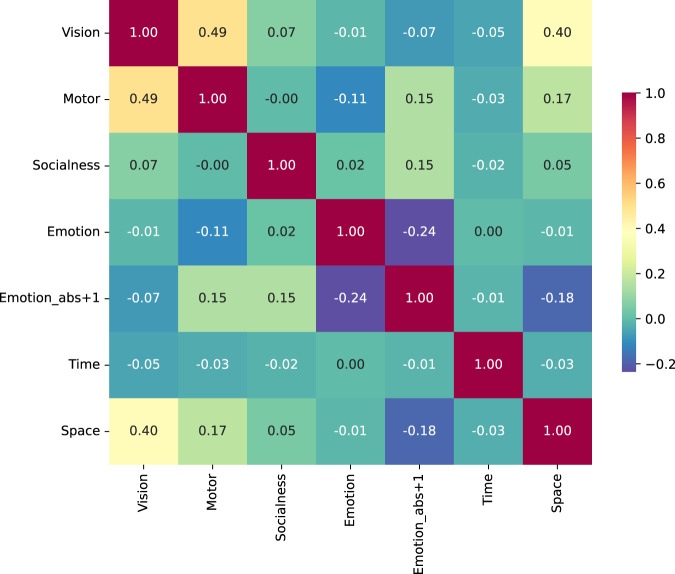
Pearson correlation coefficients between the seven semantic dimensions of ratings.

As shown in Fig. 1, the distributions of the word ratings on all dimensions for the 17940 items are skewed, indicating that for each dimension, only a small proportion of words contain rich semantic information. Because our experimental stimuli are composed of the most commonly used Chinese words, these distributions should represent Chinese vocabulary. Figure 2 shows the correlations between the seven dimensions of rating data. Most correlations are low, indicating that the semantic dimensions are mostly independent. The highest correlations were found between the dimensions Vision and Motor (r = 0.49) and Vision and Space (r = 0.40). These correlations are reasonable because the visual system plays an important role in perceiving and acquiring motor and spatial information.

### Computational extension dataset

#### Chinese

By combining the subjective rating data with computational models, we estimated the semantic ratings of a vocabulary of 1,427,992 Chinese words. This vocabulary was constructed by including the words consisting of Chinese characters and with counts no less than 5 in the Xinhua news corpus (19.7 GB in total and collected from http://www.xinhuanet.com/whxw.htm).

We first tested a variety of context-insensitive models (GloVe and Word2Vec that their word embeddings are static) and context-sensitive models (GPT2, BERT ERNIE, and MacBERT^47^ that their word embeddings vary according to their context) in predicting semantic ratings. Results show that Word2vec and MacBERT achieved the best performance in their category in the cross-validation analysis (average Pearson correlation between the predicted and actual rating scores across all dimensions: 0.613, 0.782, 0.850, 0.877, 0.881, 0.886, for GloVe, Word2Vec, GPT2, BERT, ERNIE and MacBERT). Therefore, in the following experiments, we utilized these two representative models to extract word representations for Chinese words. Specifically, for Word2vec, we used the default parameters as Skip-Gram architecture with embedding dimensions of 300. To obtain the word embeddings from MacBERT, following Chersoni *et al*.^48^, we extracted 10 to 1,000 sentences for each word (depending on the counts of the word) from the Xinhua corpus and used MacBERT to calculate the representations of the sentences. We then calculated the averaged sentence representation and used it as the word representation. We obtained 1,427,992 word representations from Word2vec and 900,243 (here, we only use words with counts greater than 10) from MacBERT.

Afterward, for each of the seven semantic dimensions and each word embedding method, we trained a ridge regression model with a 10-fold cross-validation method to learn the mapping function from word representations to the mean semantic ratings of corresponding words. We then use the best-trained regression model (which achieved the lowest error on the validation set out of 10 models from the 10-fold validation) to estimate the semantic ratings for the extended Chinese vocabulary.

#### English

We also extended the computational dataset to an English vocabulary. This extension is based on the assumption that the Chinese and English semantic spaces share the coarse-grained semantic dimensions that we studied. The assumption got direct support from the high cross-language validity of our Chinese dataset: for all semantic dimensions, the ratings of Chinese words were strongly correlated with the ratings of their English translations from previously published English rating datasets (see Technical Validation). The English vocabulary includes 1,515,633 words. This vocabulary was constructed by including the words with counts no less than 5 in the Wikipedia corpus (13 G and downloaded from https://dumps.wikimedia.org/enwiki/latest/). Consistent with the methods for constructing the extensional Chinese dataset, we utilized Word2vec with default parameters and the pretrained BERT model (which has been proven to achieve the best performance at predicting semantic features among other variations^49^) to extract word representations for each English word. Specifically, to obtain word embeddings from BERT, we first extract 10 to 1,000 sentences (depending on the word counts) from the Wikipedia corpus for each. We use BERT to calculate the sentence representations. We then use the averaged sentence representation as the word representation. We obtained 1,515,633 word representations from Word2vec and 930,668 (here, we only use words with counts more than 10) from BERT.

To estimate the semantic ratings of English words, we first trained a model to align the English embedding space to the Chinese embedding space. Specifically, we extracted all single word translation pairs (i.e., remove Chinese-English pairs in which English is more than one word) from a Chinese to English dictionary, the CC-CEDICT, at https://www.mdbg.net/chinese/dictionary?page=cc-cedict, which is the largest open-sourced Chinese to English dictionary to our knowledge, and obtained a Chinese-English bilingual lexicon of 19,424-word pairs. Next, we trained a ridge regression model to learn the mapping between the English embeddings and Chinese embeddings based on the bilingual word pairs.

Finally, the semantic ratings for the English words were estimated in two steps. First, we projected each English word representation from the English semantic space to the Chinese semantic space. Second, the projected word representation was taken as the input word representation for the semantic rating prediction models for Chinese words. Then the output of the model was taken as the estimated semantic rating for the English word.

## Data Records

The SSDD^16^ is available on the OSF repository at 10.17605/OSF.IO/N5VKE. The data are sorted into two main folders. The first main folder is “Main_Data,” in which we provided the final subjective and estimated rating results. The second main folder is called “Supplementary_Data,” in which we provided the information of participants, the instructions for the rating experiments, the raw rating data, the validation data for the subjective ratings and computational extension ratings, the word embeddings, and the code for calculating and validating the estimated ratings. More details about the data are provided below.

### Main Data

The average ratings across participants for the 17,940 Chinese words on the six rated semantic dimensions are provided in the file “Rated_semantic_dimensions.csv.” Additionally, we also provided the absolute value of the average emotion rating for each word as the seventh dimension, which is called “emotion_abs + 1” in the file. The estimated semantic ratings for extensional Chinese and English vocabularies using different computational models are provided in four files named “Estimated_semantic_dimensions_word2vec_Chinese.csv”, “Estimated_semantic_dimensions_macbert_Chinese.csv”, “Estimated_semantic_dimensions_word2vec_English.csv”, and “Estimated_semantic_dimensions_bert_English.csv”.

### Supplementary data

#### Information of participants

The file named “Information_Participants.xlsx” provides the age and sex of the participants, the number of valid and invalid sessions and words that the participants’ data contain, and which of the six experiments the participants participated in and provided valid data.

#### Instructions for the rating experiments

At the start of each session of each rating experiment, participants were provided an instruction that contained the working definition of the semantic dimension and a few examples. These instructions are provided in the file named “Instructions.docx.”

#### Raw rating data

The raw data of the 6 rating experiments are provided under the subfolder named “Raw_rating_data.” The data are sorted into six folders named by the rated dimensions (Vision, Motor, Socialness, Emotion, Time, and Space). Under each folder, 18 files (named “session*.csv,” in which * is 1 to 18) correspond to the 18 sessions. In each file, the column named “Word” provides the items for which the semantic ratings were collected, for example, ‘花朵’ (meaning “Flower” in English). The remaining columns are named by the initials of 30 participants who rated the words and show the rating scores from each participant.

#### Validation data for the subjective ratings

In the file “Validation_Ratings.xlsx,” we provided the data and results of the validation analyses for our ratings. In addition to the validation analyses and results mentioned in the section “Validity of the subjective rating dataset” for each dimension of ratings, we also provided the correlations of our ratings to several fine-grained semantic dimensions of ratings provided by Binder *et al*.^1^.

#### Validation data for the computational extension ratings

In the file “Validation_Computational_Ratings.xlsx,” we provided the data and results of the validation analyses for our computational extension dataset.

#### Word embeddings

The word vectors used to compute the computational extension datasets are provided in the subfolder named “Word embeddings,” including the Word2vec and MacBERT embeddings for Chinese words and the Word2vec and BERT embeddings for English words.

#### Code for calculating and validating the estimated ratings

See the section “Code Availability.”

## Technical Validation

### Reliability of the subjective rating dataset

We examined the reliability of the ratings by computing the intraclass correlation coefficients (ICCs) for each experiment and each session. For each experiment, we calculated the one-way random ICC because different participants rated different items; for each session, we calculated the two-way random ICC because there were always 30 consistent participants who rated all items^50,51^. The results are summarized in Tables 2, 3. For all experiments and all sessions, the ICCs were above 0.9, which indicates good reliability of the ratings. In addition, in the SSDD, we rerated the socialness of 945 words from a prior study of us^10^ and obtained a cross-study correlation of 0.955.

**Table 2:** ICCs for each experiment (One-Way Random).

Vision	Motor	Socialness	Emotion	Time	Space
0.954	0.937	0.966	0.974	0.976	0.964

**Table 3:** ICCs for each session of each experiment (Two-Way Random, consistency).

	Vision	Motor	Socialness	Emotion	Time	Space
Session1	0.979	0.981	0.962	0.975	0.980	0.977
Session2	0.975	0.976	0.957	0.973	0.981	0.962
Session3	0.965	0.972	0.956	0.974	0.973	0.979
Session4	0.969	0.976	0.961	0.973	0.974	0.979
Session5	0.967	0.968	0.961	0.974	0.979	0.976
Session6	0.972	0.972	0.961	0.973	0.976	0.980
Session7	0.970	0.973	0.961	0.972	0.977	0.976
Session8	0.971	0.969	0.952	0.971	0.974	0.980
Session9	0.972	0.972	0.953	0.970	0.975	0.974
Session10	0.969	0.971	0.956	0.971	0.975	0.981
Session11	0.969	0.970	0.956	0.971	0.977	0.980
Session12	0.970	0.968	0.954	0.968	0.973	0.979
Session13	0.970	0.972	0.958	0.969	0.974	0.976
Session14	0.968	0.968	0.958	0.970	0.975	0.983
Session15	0.971	0.968	0.959	0.969	0.975	0.984
Session16	0.971	0.970	0.943	0.967	0.975	0.980
Session17	0.970	0.969	0.947	0.970	0.975	0.981
Session18	0.971	0.970	0.952	0.966	0.975	0.983

### Validity of the subjective rating dataset

We examined the validity of the ratings by calculating the correlations between the ratings obtained in the current study and those provided in several previous studies. The results are shown in Table 4. The full set of validation data is provided in the Supplementary Data of the database.

**Table 4:** Results of the validation analysis.

Study1	Dimension1	Study2	Dimension2	df	r
Current_study (Chinese)	Vision	Binder *et al*.^1^ (English)	Vision	533	0.756
Current_study (Chinese)	Vision	Liu *et al*.^22^ (Chinese)	Imageability	1323	0.627
Current_study (Chinese)	Vision	Su *et al*.^52^ (Chinese)	Imageability	6976	0.821
Current_study (Chinese)	Motor	Binder *et al*.^1^ (English)	Motor_General	533	0.426
Current_study (Chinese)	Motor	Heard *et al*.^45^ (English)	Pantomime	207	0.806
Current_study (Chinese)	Socialness	Binder *et al*.^1^ (English)	Social	533	0.724
Current_study (Chinese)	Socialness	Diveica *et al*.^3^ (English)	Socialness	2007	0.724
Current_study (Chinese)	Emotion	Binder *et al*.^1^ (English)	Pleasant_minus_Unpleasant	533	0.795
Current_study (Chinese)	Emotion	Xu *et al*.^55^ (Chinese)	Valence	6087	0.935
Current_study (Chinese)	Emotion_abs + 1	Binder *et al*.^1^ (English)	Arousal	533	0.532
Current_study (Chinese)	Emotion_abs + 1	Tamir *et al*.^5^ (English)	Emotion	164	0.617
Current_study (Chinese)	Emotion_abs + 1	Xu *et al*.^55^ (Chinese)	Arousal	6087	0.585
Current_study (Chinese)	Time	Binder *et al*.^1^ (English)	Time_General	533	0.715
Current_study (Chinese)	Space	Binder *et al*.^1^ (English)	Space_General	533	0.716

The language of the stimuli rated in each study is indicated in parentheses. Note: The following dimensions are calculated based on the original ratings from Binder *et al*.^1^. 1) The scores of Motor_General are calculated by averaging the ratings of the dimensions belonging to the domain of Motor, which include Head, UpperLimb, LowerLimb, and Practice. 2) The scores of Pleasant_minus_Unpleasant are calculated by subtracting the ratings of Unpleasant from those of Pleasant. 3) The scores of Time_General are calculated by averaging the ratings of Time and Duration. 4) The scores of Space_General are calculated by averaging the ratings of the dimensions belonging to the domain of Spatial, which include Landmark, Path, Scene, Near, Toward, and Away.

For the semantic dimension of vision (visual imageability), the ratings were validated based on Binder *et al*.^1^, Liu *et al*.^22^, and Su *et al*.^52^. The rating instructions used in the 4 studies are similar. Binder *et al*.^1^, Liu *et al*.^22^, and Su *et al*.^52^ obtained their ratings using English words, single-character Chinese words, and two-character Chinese words, respectively. Their correlations to the current study are 0.756, 0.627, and 0.821. The relatively lower correlation to Liu *et al*.^22^ than to Su *et al*.^52^ might be due to that many Chinese characters have multiple meanings so that single-character words are more often ambiguous in their semantics than two-character words.

For the semantic dimension of motor, the ratings were validated based on Heard *et al*.^45^ and Binder *et al*.^1^. The rating instructions used in the current study and Heard *et al*.^45^ are very similar, both focusing on how easily a word’s referent can be pantomimed. Similar motor-semantic ratings have been used to reflect the general impact of motor-semantic representation on cognition and neural activities in several previous studies^18,44,46,53,54^. The correlation between Heard *et al*.^45^ and the current study is 0.806. Binder *et al*.^1^ did not set any general rating for the motor dimension. We therefore correlated our ratings with the four fine-grained motor ratings of Binder *et al*.^1^, i.e., Head, UpperLimb, LowerLimb, and Practice. The correlations are in the range of 0.133 to 0.342. We further correlated our ratings with the mean of the four motor ratings and obtained a correlation of 0.426. These relatively low correlations indicate that the four motor dimensions rated by Binder *et al*.^1^ may not be able to fully explain the content of our ratings. It is likely that our ratings reflect more dimensions of motor knowledge than those included in Binder *et al*.^1^, such as postures and gestures. For example, the word ‘怀孕’ meaning “pregnant” was rated as high-socialness. Being pregnant is associated with specific postures and whole-body motor features but not with specific motor features of the head, feet, or hands. In addition, people often use gestures to represent some particular concepts, especially when performing pantomimes or playing charades.These gestures should also be viewed as a type of motor knowledge as long as people can reach a consensus on their meanings. The motor-rating instructions used in Heard *et al*.^45^ and the current study should be more sensitive in detecting these additional types of motor knowledge than those used by Binder *et al*.^1^.

For the semantic dimension of socialness, the ratings were validated based on Diveica *et al*.^3^ and Binder *et al*.^1^. The core ideas of the instructions used in the 3 studies are all centered on interpersonal interactions and relationships. However, the instructions used in the current study and Binder *et al*.^1^ were both brief, while those used by Diveica *et al*.^3^ were much more detailed, that is, “a social characteristic of a person or group of people, a social behavior or interaction, a social role, a social space, a social institution or system, a social value or ideology, or any other socially relevant concept.” The correlations of our ratings to those of Diveica *et al*.^3^ and Binder *et al*.^1^ are both 0.724.

For the semantic dimension of emotion (valence), the ratings were validated based on Xu *et al*.^55^ and Binder *et al*.^1^. The instructions used in the current study and Xu *et al*.^55^ are similar, and the correlation between the two studies is 0.935. The emotion ratings of the current study are closely associated with two dimensions of Binder *et al*.^1^, that is Pleasant and Unpleasant. Therefore, we calculated composite scores of the two dimensions by subtracting the ratings of Unpleasant from those of Pleasant and correlated the scores with our ratings. The correlation is 0.795.

We also validated the absolute values of our emotion ratings. As mentioned above, this measure, which can be referred to as the dimension of “valenced vs. neutral”, can reflect the relatedness of word meanings to emotion. To validate this measure, we correlate the absolute values with the emotion ratings collected by Tamir *et al*.^5^. The correlation is 0.617, indicating that the absolute values of our emotion ratings can reflect the general emotional relatedness of words. Additionally, the absolute value of valence is also related to another important dimension of emotion, called arousal. Arousal increases as a function of both positive and negative valence^56^. The absolute value of valence rating has been used to represent arousal in some previous studies^57^. We correlated the absolute values of our emotion ratings with the arousal ratings provided in Xu *et al*.^55^ and Binder *et al*.^1^. The correlations are 0.585 and 0.532, respectively, which is consistent with the findings in the literature.

Finally, for the semantic dimensions of time and space, we validated the ratings based on Binder *et al*.^1^. Binder *et al*.^1^ did not set any general rating for these dimensions. Therefore, we averaged the ratings of two time-related dimensions (Time and Duration) to correspond to our time ratings and averaged the ratings of six space-related dimensions (Landmark, Path, Scene, Near, Toward, and Away) to correspond to our space ratings. The correlations are 0.715 and 0.716 for time and space ratings, respectively.

The validation results indicate good validity of our ratings. They also indicate that the semantic ratings of the current study can be to a large extent generalized from Chinese to English. As shown in Table 4, for all 6 original ratings, the cross-language correlations can reach above 0.7. The only low correlation is with the “Motor_General” measure of Binder *et al*.^1^, which has been explained above. The absolute values of our emotion ratings also have a correlation of 0.617 with the emotion ratings of Tamir *et al*.^5^. These cross-language correlations are close to some of the reported correlations between English studies. For example, as reported by Diveica *et al*.^3^, their correlation with Binder *et al*.^1^ is 0.76, which is only slightly higher than ours. Therefore, although it should be noted that language and cultural differences should have impacts on semantic ratings, there is still high cross-language validity of our ratings to support computational estimations on English words.

### Validity of the computational extension dataset

We conducted two validation analyses to test the validity of the estimated ratings for the extensional vocabulary. The first analysis aimed to examine the internal validity of the outputs of our computational predictive models. To this end, we calculated the average correlation between the predicted and actual ratings for Chinese words across the 10-fold cross-validation training. Specifically, the 10-fold cross-validation training first split all dataset into 10 folds, then used 9 folds to train the model and the predicted semantic ratings for words in the left one fold, then calculated the correlation of the predicted semantic ratings with the actual ratings to evaluate the model. The results are shown in Table 5. For both word embedding methods, the models performed well for all semantic dimensions, indicating good internal validity.

**Table 5:** Results of the cross-validation analysis for the estimated ratings of Chinese words.

	Vision	Motor	Socialness	Emotion	Emotion_abs + 1	Time	Space
Word2vec (Chinese)	0.849	0.755	0.829	0.773	0.749	0.694	0.823
MacBERT (Chinese)	0.913	0.836	0.895	0.894	0.861	0.892	0.910

The second analysis aimed to examine the external validity of the estimated/predicted ratings for the extended Chinese and English words. To this end, we conducted a validation analysis following the same method that we used for validating the subjective ratings. The results are shown in Tables 6–9. For all dimensions, the estimated/predicted ratings of all models showed good external validity, which is very close to that of the subjective ratings (see Table 4).

**Table 6:** Results of the validation analysis for the estimated ratings of Chinese words by Word2vec.

Study1	Dimension1	Study2	Dimension2	df	r
Word2vec (Chinese)	Vision	Binder *et al*.^1^ (English)	Vision	517	0.700
Word2vec (Chinese)	Vision	Liu *et al*.^22^ (Chinese)	Imageability	1323	0.511
Word2vec (Chinese)	Vision	Su *et al*.^52^ (Chinese)	Imageability	6975	0.755
Word2vec (Chinese)	Motor	Binder *et al*.^1^ (English)	Motor_General	517	0.479
Word2vec (Chinese)	Motor	Heard *et al*.^45^ (English)	Pantomime	206	0.634
Word2vec (Chinese)	Socialness	Binder *et al*.^1^ (English)	Social	517	0.635
Word2vec (Chinese)	Socialness	Diveica *et al*.^3^ (English)	Socialness	2003	0.682
Word2vec (Chinese)	Emotion	Binder *et al*.^1^ (English)	Pleasant_minus_Unpleasant	517	0.741
Word2vec (Chinese)	Emotion	Xu *et al*.^55^ (Chinese)	Valence	6087	0.828
Word2vec (Chinese)	Emotion_abs + 1	Binder *et al*.^1^ (English)	Arousal	517	0.563
Word2vec (Chinese)	Emotion_abs + 1	Tamir *et al*.^5^ (English)	Emotion	159	0.607
Word2vec (Chinese)	Emotion_abs + 1	Xu *et al*.^55^ (Chinese)	Arousal	6087	0.573
Word2vec (Chinese)	Time	Binder *et al*.^1^ (English)	Time_General	517	0.603
Word2vec (Chinese)	Space	Binder *et al*.^1^ (English)	Space_General	517	0.666

**Table 7:** Results of the validation analysis for the estimated ratings of Chinese words by MacBERT.

Study1	Dimension1	Study2	Dimension2	df	r
MacBERT (Chinese)	Vision	Binder *et al*.^1^ (English)	Vision	532	0.713
MacBERT (Chinese)	Vision	Liu *et al*.^22^ (Chinese)	Imageability	1323	0.505
MacBERT (Chinese)	Vision	Su *et al*.^52^ (Chinese)	Imageability	6976	0.796
MacBERT (Chinese)	Motor	Binder *et al*.^1^ (English)	Motor_General	532	0.444
MacBERT (Chinese)	Motor	Heard *et al*.^45^ (English)	Pantomime	207	0.689
MacBERT (Chinese)	Socialness	Binder *et al*.^1^ (English)	Social	532	0.679
MacBERT (Chinese)	Socialness	Diveica *et al*.^3^ (English)	Socialness	2007	0.712
MacBERT (Chinese)	Emotion	Binder *et al*.^1^ (English)	Pleasant_minus_Unpleasant	532	0.781
MacBERT (Chinese)	Emotion	Xu *et al*.^55^ (Chinese)	Valence	6087	0.894
MacBERT (Chinese)	Emotion_abs + 1	Binder *et al*.^1^ (English)	Arousal	532	0.549
MacBERT (Chinese)	Emotion_abs + 1	Tamir *et al*.^5^ (English)	Emotion	164	0.681
MacBERT (Chinese)	Emotion_abs + 1	Xu *et al*.^55^ (Chinese)	Arousal	6087	0.577
MacBERT (Chinese)	Time	Binder *et al*.^1^ (English)	Time_General	532	0.668
MacBERT (Chinese)	Space	Binder *et al*.^1^ (English)	Space_General	532	0.607

**Table 8:** Results of the validation analysis for the estimated ratings of English words by Word2Vec.

Study1	Dimension1	Study2	Dimension2	df	r
Word2vec (English)	Vision	Binder *et al*.^1^ (English)	Vision	531	0.719
Word2vec (English)	Motor	Binder *et al*.^1^. (English)	Motor_General	531	0.405
Word2vec (English)	Motor	Heard *et al*.^45^ (English)	Pantomime	207	0.701
Word2vec (English)	Socialness	Binder *et al*.^1^ (English)	Social	531	0.597
Word2vec (English)	Socialness	Diveica *et al*.^3^ (English)	Socialness	2007	0.704
Word2vec (English)	Emotion	Binder *et al*.^1^ (English)	Pleasant_minus_Unpleasant	531	0.766
Word2vec (English)	Emotion_abs + 1	Binder *et al*.^1^ (English)	Arousal	531	0.557
Word2vec (English)	Emotion_abs + 1	Tamir *et al*.^5^ (English)	Emotion	161	0.552
Word2vec (English)	Time	Binder *et al*.^1^ (English)	Time_General	531	0.640
Word2vec (English)	Space	Binder *et al*.^1^ (English)	Space_General	531	0.685

**Table 9:** Results of the validation analysis for the estimated ratings of English words by MacBERT.

Study1	Dimension1	Study2	Dimension2	df	r
BERT (English)	Vision	Binder *et al*.^1^ (English)	Vision	531	0.691
BERT (English)	Motor	Binder *et al*.^1^ (English)	Motor_General	531	0.475
BERT (English)	Motor	Heard *et al*.^45^ (English)	Pantomime	207	0.688
BERT (English)	Socialness	Binder *et al*.^1^ (English)	Social	531	0.624
BERT (English)	Socialness	Diveica *et al*.^3^ (English)	Socialness	2007	0.720
BERT (English)	Emotion	Binder *et al*.^1^ (English)	Pleasant_minus_Unpleasant	531	0.779
BERT (English)	Emotion_abs + 1	Binder *et al*.^1^ (English)	Arousal	531	0.541
BERT (English)	Emotion_abs + 1	Tamir *et al*.^5^ (English)	Emotion	161	0.671
BERT (English)	Time	Binder *et al*.^1^ (English)	Time_General	531	0.656
BERT (English)	Space	Binder *et al*.^1^ (English)	Space_General	531	0.701

Note that the semantic ratings in the computational extension dataset are calculated using distributional language models, which may not give ratings as accurate as human annotations. Existing work has proven that word embeddings from Word2vec, BERT and other language models encode rich semantic information. However, it is unclear what exact semantic feature it encodes; that is, word embeddings may not encode all information in the semantic dimensions of vision, motor, socialness, emotion, time, and space. Moreover, word embeddings are calculated by counting word cooccurrence in a large corpus, which is different from how humans learn and understand word meaning. Therefore, the computational extension dataset may show different patterns than human annotated data. Furthermore, the mismatch between English and Chinese semantic spaces is a potential limitation of our method because we projected English words from the English semantic space to the Chinese semantic space to accomplish our estimation.

## Data Availability

The codes for calculating and evaluating computational extension scores are available in the subfolder named “Code” under the folder named “Supplimentary_Data” at 10.17605/OSF.IO/N5VKE^16^. Specifically, to estimate the semantic ratings of Chinese words, we first used “train_decode.py” to learn a mapping function from the Chinese embedding space to semantic ratings based on the 17,940 subjective ratings with their corresponding word embeddings. We then utilized “predict.py” to generate the semantic ratings of all extensional Chinese words. To estimate the semantic ratings for English words, we need to align the mapping relations between the English and Chinese embedding spaces beforehand. To achieve that, we first used “extract_en.py” and “extract_zh.py” to extract word representations that are in the Chinese-English bilingual lexicon and then used “match.py” and “train_align.py” to learn the mapping function from English to Chinese word representations. Finally, based on the two mapping functions, including English-Chinese mapping and Chinese to semantic ratings, we used “predict.py” to project English embedding peace to that of Chinese to generate the semantic ratings of all extensional English words. To validate the above results, we used “corr_binder.py” and “corr_binder_cn.py” to compute correlations between the extensional ratings and corresponding scores in Binder *et al*.^1^.
