# Author Correction: A large dataset of semantic ratings and its computational extension

**DOI:** 10.1038/s41597-023-02479-3

**Published:** 2023-08-22

**Authors:** Shaonan Wang, Yunhao Zhang, Weiting Shi, Guangyao Zhang, Jiajun Zhang, Nan Lin, Chengqing Zong

**Affiliations:** 1 National Laboratory of Pattern Recognition, Institute of Automation, CAS, Beijing, China; 2 School of Artificial Intelligence, University of Chinese Academy of Sciences, Beijing, China; 3 CAS Key Laboratory of Behavioural Sciences, Institute of Psychology, Beijing, China; 4 Department of Psychology, University of Chinese Academy of Sciences, Beijing, China

**Keywords:** Human behaviour, Language, Human behaviour, Language

Correction to: *Scientific Data* 10.1038/s41597-023-01995-6, published online 23 February 2023

In this article the funding from Youth Innovation Promotion Association CAS was omitted. The original article has been corrected.

